# Prevalence and risk factors of periodontal disease among pre-conception Chinese women

**DOI:** 10.1186/s12978-016-0256-3

**Published:** 2016-12-01

**Authors:** Hong Jiang, Yi Su, Xu Xiong, Emily Harville, Hongqiao Wu, Zhijun Jiang, Xu Qian

**Affiliations:** 1 School of Public Health, Key Laboratory of Public Health Safety, Ministry of Education, Fudan University, Mailbox 175, No. 138 Yixueyuan Road, Shanghai, 200032 People’s Republic of China; 2 Oral Health Department, Eye & ENT Hospital of Fudan University, 19 Baoqing Road, Shanghai, 200032 China; 3 School of Public Health and Tropical Medicine, Tulane University, 1440 Canal Street, New Orleans, LA 70112 USA; 4 Reproductive Health Department, Maternal and Child Health Care Hospital, Changzhou Municipality, 16 Boai Road, Changzhou, Jiangsu Province 200032 China; 5 Oral Health Department, Maternal and Child Health Care Hospital, Changzhou Municipality, 16 Boai Road, Changzhou, Jiangsu Province 200032 China; 6 Global Health Institute, School of Public Health, Fudan University, Mailbox 175, No. 138 Yixueyuan Road, Shanghai, 200032 China

**Keywords:** Periodontal disease, Prevalence, Risk factors, Pre-conception women

## Abstract

**Background:**

Periodontal disease is one of the most common chronic infectious diseases. It has been reported that periodontal disease is associated with various adverse pregnancy outcomes including preterm birth, low birth weight, and gestational diabetes mellitus. Given the fact that the treatment for periodontal disease during pregnancy was ineffective in improving pregnancy outcomes by most of studies, the pre-conception period has been put forward as a more optimal time. However, very few studies have reported the prevalence of periodontal disease among pre-conception women. This study aimed to examine the prevalence and risk factors of periodontal disease among Chinese pre-conception women.

**Methods:**

A survey was conducted among pre-conception women at the Maternal and Child Health Hospital, Changzhou, China between January 2012 and December 2014. A total of 987 pre-conception women were recruited for a full-mouth dental examination after providing informed consent. A dental examination was carried out by probing six sites per tooth using a manual UNC-15 probe and a recording form.

**Results:**

The overall rate of periodontal disease among participants was 73.9% (729/987) (95% confidence interval (CI): 71.0–76.6%). Among women with periodontal disease, 48.0% of cases were mild, 50.9% were moderate and 1.1% were severe. Self-reported bleeding during tooth brushing was the only significant predictive factor for overall periodontal disease (adjusted odds ratio (aOR): 3.71, 95% CI: 2.24, 6.15, *P* < 0.001) and moderate/severe periodontal disease (aOR: 5.17, 95% CI: 3.05, 8.79, *P* < 0.001).

**Conclusion:**

A high prevalence of periodontal disease was found in pre-conception Chinese women. Women who have bleeding during tooth brushing could be at increased risk of periodontal disease, and might require further oral health care.

## Background

Periodontal disease is an inflammatory condition of the soft tissues surrounding the teeth and the gradual destruction of the supporting structures of the teeth [1, 2]. Periodontal disease is one of the most common chronic infectious diseases, with the overall prevalence from 10 to 90% in adults [3–5], depending on diagnostic criteria. Risk factors include the increasing age, male gender, some ethnicities, low educational level, poor economic status, tobacco use, psychological distress, and poor oral hygiene [5–9]. The increased gestational age was also a risk factor. Periodontal disease is associated with systemic diseases such as cardiovascular diseases and diabetes [10, 11], as well as various adverse pregnancy outcomes including preterm birth, low birth weight, early pregnancy loss, gestational diabetes mellitus and preeclampsia [12–14]. Among pregnant women, the prevalence of periodontal disease is also high, ranging from 10 to 74% [6, 9]. Due to the high prevalence of periodontal disease and its association with systemic disease, much more attention has been attracted from both the medical and public health areas, in order to improve the health of the whole population.

Most of previous studies have been focusing on studying the prevalence of periodontal disease during the time period of pregnancy and its impact on pregnancy outcomes [9, 11–13]. It is well known that pregnancy itself increases the prevalence of gingivitis and periodontitis [15, 16]. However, little is known about the prevalence and risk factors among pre-conception women who plan to conceive. In addition, given the fact that several large clinic trials demonstrated the treatment for periodontal disease during pregnancy was ineffective in improving pregnancy outcomes [17–19], and treating periodontal disease during pregnancy potentially leads to an increase in bacteremia, which itself may increase the risk of adverse pregnancy outcomes, it has been put forward that the pre-conception period may be a more optimal time for the treatment of periodontal disease [20, 21]. It is important to understand the prevalence and risk factors of periodontal disease among women during pre-conception period. This paper aims to report the prevalence and severity of periodontal disease and its risk factors among pre-conception women.

## Methods

Data for this paper were drawn from the baseline survey of women enrolled in a randomized controlled trial for treating periodontal disease in pre-conception women to improve periodontal status during pregnancy and birth outcomes (Trial registration No.: ChiCTR-TRC-12001913) [21]. Ethical approval of the research was granted by the Institutional Review Boards of the School of Public Health, Fudan University, Shanghai, China and Tulane University, New Orleans, USA, and written informed consent was obtained from each participant before participation.

### Participants and recruitment

The baseline survey was conducted between January 2012 and December 2014 at the Maternal and Child Health Hospital, in Changzhou Municipality, a city in eastern China, with a population of approximately 4.6 million. It is the only maternal and child health care hospital in the city, with a total of 9,000 deliveries annually, covering more than 70% of deliveries in the city [21].

Women were invited to participate in the study when they attended the pre-conception health care clinic at the hospital. They were approached by research nurse who introduced the study and asked about their willingness to participate. After women agreed to join, they were assessed for the eligibility. Eligible women first filled in a baseline questionnaire including demographic information such as age, education level, employment status, family monthly income, and periodontal disease relevant information such as oral hygiene, health insurance status, family history of periodontal disease and stress. Women were then provided a free full-mouth dental examination.

Detailed inclusion and exclusion criteria are listed in the study protocol [21]. Overall, the inclusion criteria included the desire to become pregnant within one year, 18 years or older, and planning to deliver at the recruiting hospital. The exclusion criteria included being younger than 18 years old, having fewer than 20 teeth, contraindication to probing in a dental examination, being unwilling or unable to sign the informed consent form, receiving periodontal treatment within the past six months, or changing the plan to become pregnant within a year.

### Sample size estimation

Given the margin of error 3.5%, expected rate of periodontal disease among preconception women 55% (estimated from the pilot study) [21], the sample size of 777 women was adequate to estimate the prevalence with 95% confidence interval. Taking into account the loss to follow-up around 20% between preconception period and childbirth, we expanded the recruitment of around 1000 women in the baseline survey for a dental examination.

### Definition and measurement of periodontal disease

The oral examination for periodontal disease was carried out by probing six sites per tooth (mesio-buccal, mesio-lingual, disto-buccal, disto-lingual, mid-buccal and mid-lingual), using a manual UNC-15 probe and a record form [12]. All examination was completed by a single dentist in the hospital and the data were recorded by a trained nurse. Prior to the study, a calibration section was conducted. Three volunteers were examined by the dentist and an experienced periodontist simultaneously. They discussed any differences in measurement and the measurement approach was adjusted until a final agreement was reached.

There is no universally accepted research definition for the diagnosis of periodontal disease. Usual indicators combine probing depth (the distance in millimeters from the gingival margin to the apical part of the pocket, PD) and clinical attachment loss (the distance between the cemento-enamel junction and the base of the pocket, CAL) over a certain threshold [1, 21]. In this study, the definition of periodontal disease referred to the study by Offenbacher et al. published in 2001 [22]. The overall periodontal disease was defined as a presence of any site exhibiting PD > 3 mm or CAL > 3 mm. The moderate periodontal disease was defined as the presence of 4 or more sites with PD > 3 mm. Severe periodontal disease was defined as 4 or more sites with PD ≥ 5 mm. For comparison with other studies, we also calculated the prevalence of periodontal disease based on the definitions by Arbes et al. and the U.S. National Health and Nutrition Examination Survey (NHANES) in 2009 and 2010 [5, 23]. The definition of periodontal disease by Arbes, et al. was 1 or more sites with PD ≥ 4 mm and CAL ≥ 3 mm [23]. In the NHANES of 2009 and 2010, the definition of mild periodontitis was 2 or more sites with PD ≥ 4 mm and CAL ≥ 3 mm (not on the same tooth). Moderate periodontitis was 2 or more sites with PD ≥ 5 mm and CAL ≥ 4 mm (not on the same tooth). Severe periodontitis was 1 or more sites with PD ≥ 5 mm and 2 or more sites with CAL ≥ 6 mm (not on the same tooth) [5].

### Data analysis

Women’s Body Mass Index (BMI) was calculated as weight (kg)/height^2^ (metres^2^). Overweight was defined as BMI ≥ 25 [24]. The frequency of daily tooth brushing was based on self-reported oral hygiene behavior. Response to the question such as “at least two times of tooth brushing per day” was coded as one point, otherwise the response received zero points. Women’s stress was measured using the Chinese version of the Perceived Stress Scale [25]. The scale contains 14 items, among which scores of seven items were reverse-coded; total scores were obtained by summing across all 14 items. Based on the median scores women were categorized into the “high” (equal or above the median) or the “low” stress groups (below the median).

All data were double-entered using Epidata 3.1 and checked for correctness. A Pearson Chi-Square test was used for categorical outcomes. Multiple logistic regression was performed to determine the factors associated with overall periodontal disease and moderate/severe (combined) periodontal disease. The adjusted odds ratio (OR) and its 95% confidence interval (CI) were derived from the coefficients of the logistic models and their standard errors. Statistical analyses were carried out using the Statistical Package for Social Sciences (SPSS) for Windows version 17.0.

## Results

A total of 987 pre-conception women participated in the baseline questionnaire survey and completed the dental examination. The main characteristics of pre-conception women in this study are shown in Table 1. The mean age of the participants was 27 years old (range 18–40 years). Most participants (87.2%) had college or above education level. Overall 78.6% of participants were employed and 95.8% reported monthly family income was above 4,000 RMB (~USD $615, the minimum monthly income criterion). The majority of women (85.4%) were nulliparous. 13.2% women reported at least one of their family members (including mother, father, siblings or other relatives e.g. aunty, uncle) had periodontal disease. Only 6.2% of women could get complete or partial reimbursement for oral health care from their health insurance. 17.9% of women reported to always or often having bleeding during daily tooth brushing. 40.1% (396/987) women had their last visit to a dentist within past two years, 27.1% (267/987) women had visited a dentist more than 2 years previously; and 32.8% (324/987) had never visited a dentist.

**Table 1:** Characteristics of participants and rate of periodontal disease and relevant indicators (*n* = 987)

Characteristics	*n* (%)
Age	
< 30	796 (80.6)
≥ 30	191 (19.4)
Education level	
Junior high school and below	54 (5.5)
Senior high school	72 (7.3)
College and above	861 (87.2)
Family income per month	
< 4,000RMB	41 (4.2)
≥ 4,000RMB	946 (95.8)
Employment status	
Unemployed	211 (21.4)
Employed	776 (78.6)
Overweight	
No	912 (92.4)
Yes	75 (7.6)
Periodontitis family history	
No	464 (47.0)
Yes	130 (13.2)
unknown	393 (39.8)
Parity	
0	843 (85.4)
1 or 2	144 (14.6)
Health insurance for oral health	
No	926 (93.8)
Yes	61 (6.2)
Oral hygiene	
Tooth brushing at least 2 times per day	183 (18.5)
Tooth brushing less than 2 times per day	804 (81.5)
Stress	
Lower	443 (44.9)
Higher	544 (55.1)
Self-reported bleeding during tooth brushing	
Occasionally or never	810 (82.1)
Always or often	177 (17.9)
Overall rate of periodontal disease n (%)	729 (73.9)
Any site with bleeding of probing (%)	809 (82.0)
Percentage of bleeding of probing (Median)	5.4%
Average PD Mean ± SD (mm)	1.52 ± 0.12
Average CAL Mean ± SD (mm)	1.97 ± 0.23

The overall rate of periodontal disease among participants was 73.9% (729/987) (95% CI: 71.0%-76.6%). Among women with periodontal disease, 48.0% (350/729) of cases were mild, 50.9% (371/729) were moderate, and 1.1% (8/729) were severe. The rate of at least one site of bleeding on probing was 82.0% (178/987). The median percentage of sites with bleeding on probing was 5.4%. The average PD and CAL was around 1.5 mm (SD 0.1 mm) and 2.0 (SD 0.2 mm) respectively (Table 1). When applying the definition of periodontal disease by Arbes et al., the overall rate of periodontal disease was 35.5% (350/987). Based on the definition of NHANES 2009 and 2010, the overall prevalence of periodontal disease was 31.0% (306/987), with the mild, moderate and severe cases accounting for 95.4% (292/306), 3.3% (10/306) and 1.3% (4/306) respectively.

In multivariate analyses, the only factor associated with overall periodontal disease or moderate/severe periodontal disease was self-reported bleeding during daily tooth brushing (Tables 2 and 3). Women who reported with often or always bleeding during daily tooth brushing had a higher rate of overall periodontal disease (OR 3.71, 95% CI: 2.24, 6.15, *P* < 0.001) and moderate/severe periodontal disease (OR: 5.17, 95% CI: 3.05, 8.79, *P* < 0.001) respectively, as compared to women who reported occasionally or no bleeding during tooth brushing. Overweight women were also more likely to have periodontal disease (80% vs. 73% overall, 69% vs. 57% moderate vs. severe), although this did not reach statistical significance.

**Table 2:** Factors associated with overall periodontal disease (*n* = 987)

Characteristics	Periodontitis					
	Yes *n* (%)	No *n* (%)	*P* ^*^	Adjusted OR^**^	95% CI^**^	*P* ^**^
Age (years)						
< 30	586 (80.4)	210 (81.4)	0.724	1		
≥ 30	143 (19.6)	48 (18.6)		1.07	0.68–1.68	0.767
Education level						
Junior high school and below	43 (5.9)	11 (4.3)	0.611	1		
Senior high school	53 (7.3)	19 (7.4)		0.69	0.29–1.64	0.402
College and above	633 (86.8)	228 (88.4)		0.68	0.33–1.39	0.292
Family income per month						
< 4,000RMB	31 (4.25)	10 (3.9)	0.795	1		
≥ 4,000RMB	698 (75.7)	248 (96.1)		0.86	0.41–1.83	0.695
Employment status						
Unemployed	149 (20.4)	62 (24.0)	0.226	1		
Employed	580 (79.6)	196 (76.0)		1.25	0.88–1.77	0.210
Overweight						
No	669 (91.8)	243 (94.2)	0.208	1		
Yes	60 (8.2)	15 (5.8)		1.57	0.87–2.85	0.136
Periodontitis family history						
No	344 (47.2)	120 (46.5)	0.910	1		
Yes	94 (12.9)	36 (14.0)		0.83	0.53–1.29	0.404
unknown	291 (39.9)	102 (39.5)		0.93	0.68–1.27	0.639
Parity						
0	621 (85.2)	222 (86.0)	0.736	1		
1 or 2	108 (14.8)	36 (14.0)		1.07	0.64–1.78	0.809
Health insurance for oral health						
No	683 (93.7)	243 (94.2)	0.776	1		
Yes	46 (6.3)	15 (5.8)		1.05	0.56–1.93	0.890
Oral hygiene						
Tooth brushing less than 2 times/day	135 (18.5)	48 (18.6)	0.976	1		
Tooth brushing at least 2 times/day	594 (81.5)	210 (81.4)		1.10	0.75–1.60	0.639
Stress						
Lower	329 (45.1)	114 (44.2)	0.793	1		
Higher	400 (54.9)	144 (55.8)		0.91	0.68–1.22	0.533
Self-reported bleeding during tooth brushing						
Occasionally or never	571 (78.3)	239 (92.6)		1		
Always or often	158 (21.7)	19 (7.4)	<0.001	3.71	2.24–6.15	<0.001

^*^Pearson chi-square test; ^**^Multiple logistic regression, adjusted for all other variables in the table

**Table 3:** Factors associated with moderate and severe periodontal disease compared with no periodontal disease (*n* = 637)

Characteristics	Moderate and severe periodontitis					
	Yes *n* (%)	No *n* (%)	*P* ^*^	OR^**^	95% CI^**^	*P* ^**^
Age (years)						
< 30	314 (82.8)	210 (81.4)	0.637	1		
≥ 30	65 (17.2)	48 (18.6)		0.88	0.52–1.50	0.636
Education level						
Junior middle school and below	21 (5.5)	11 (4.3)	0.690	1		
Senior middle school	24 (6.3)	19 (7.4)		0.64	0.24–1.74	0.386
College and above	334 (88.1)	228 (88.4)		0.75	0.33–1.69	0.486
Family income per month						
< 4,000RMB	12 (3.2)	10 (3.9)	0.630	1		
≥ 4,000RMB	367 (96.8)	248 (96.1)		1.11	0.45–2.71	0.827
Employment status						
Unemployed	83 (21.9)	62 (24.0)	0.529	1		
Employed	296 (78.1)	196 (76.0)		1.12	0.76–1.67	0.569
Overweight						
No	345 (91.0)	243 (94.2)	0.142	1		
Yes	34 (9.0)	15 (5.8)		1.72	0.89–3.30	0.105
Periodontitis family history						
No	178 (47.0)	120 (46.5)	0.533	1		
Yes	42 (11.1)	36 (14.0)		0.71	0.42–1.20	0.201
unknown	159 (42.0)	102 (39.5)		0.94	0.66–1.35	0.738
Parity						
0	325 (85.8)	222 (86.0)	0.917	1		
1 or 2	54 (14.2)	36 (14.0)		1.15	0.62–2.11	0.658
Health insurance for oral health						
No	358 (94.5)	243 (94.2)	0.884	1		
Yes	21 (5.5)	15 (5.8)		0.78	0.38–1.62	0.51
Oral hygiene						
Tooth brushing less than 2 times/day	80 (21.1)	48 (18.6)	0.439	1		
Tooth brushing at least 2 times/day	299 (78.9)	210 (81.4)		0.88	0.58–1.35	0.561
Stress						
Lower	174 (45.9)	114 (44.2)	0.668	1		
Higher	205 (54.1)	144 (55.8)		0.82	0.59–1.16	0.260
Self-reported bleeding during tooth brushing						
Occasionally or never	275 (72.6)	239 (92.6)		1		
Always or often	104 (27.4)	19 (7.4)	<0.001	5.17	3.05–8.79	<0.001

^*^Pearson chi-square test; ^**^Multiple logistic regression, adjusted for all other variables in the table

## Discussion

The study found a high prevalence of periodontal disease among pre-conception women despite their relatively young age. Self-reported frequent bleeding during daily tooth brushing was significantly predicting factor for developing overall periodontal disease, as well as moderate and severe periodontal disease.

The prevalence of periodontal disease in our study population was comparable to other China studies. Two surveys indicated the rates of periodontal disease nearly 50% and 78% among pregnant women of Shanghai and Shenzhen, Guangdong China respectively [6, 26]. Another survey conducted among childbearing age women in Zhejiang Province, China showed the prevalence nearly 85% [27]. All these studies indicated the high prevalence of periodontal disease among Chinese women of childbearing age. The prevalence among our study population was quite high compared to the rate of periodontal disease (approximately 20%) among non-pregnant women of childbearing age shown by NHANES III of USA [28]. However, this could be due to the different approaches of dental examination and different diagnostic criteria for periodontal disease between two studies [28]. The oral health examination in NHANES III was not a full mouth examination, but performed in two randomly chosen quadrants, one midbuccal and one mesiobuccal. Only two sites of each tooth were examined for each participant. Compared with full mouth examination, this approach has been shown to result in an underestimate of rate of periodontal disease [5, 28]. The prevalence among the pre-conception women in our study was still higher, when applying the same periodontal disease definition as the study by Arbes et al., in which the prevalence of periodontal disease was 8.8% among 18 year olds and above [23]. Furthermore, compared with the US prevalence rate around 24% in adults 30 to 34 years age in NHANES 2009 and 2010 [5], the prevalence of periodontal disease among our study participants was also higher when applying the same definition of periodontal disease and despite of the younger average age. The high prevalence of periodontal disease among pre-conception women may be due to the fact that oral health care is generally not available in China: our data also showed nearly 60% of pre-conception women visited a dentist more than two years ago or had never visited a dentist, and only around 6% of women could get complete or partial reimbursement for oral health care from their health insurance.

Nearly 50% of periodontal disease cases in our study were found to have mild periodontal disease, which indicating a need for timely oral health care to avoid further progression, as more than 50% of women with periodontal disease were moderate or severe, demonstrating the neglected oral health care among this population. Given the fact that periodontal disease often advances and becomes more severe during pregnancy due to the elevated levels of estrogen and progesterone [16, 29], the screening and treatment of periodontal disease should be included in pre-conception health care.

Periodontal disease has been reported to be more common in deprived or disadvantaged populations [9, 27]. Additional risk factors for periodontal disease include older age, Hispanic and non-Hispanic Black ethnicity, being overweight and obese, poor oral hygiene, more stressful psychological status, tobacco use, alcohol consumption, taking oral contraceptives, increased parity, gestational age, public dental insurance (versus private), and lacking a recent dental visit (within six months) [7, 9, 30, 31]. Although we have examined these potential associated factors in the analysis, the findings did not show socioeconomic status or other factors, except self-reported bleeding during tooth brushing, to be significantly associated with periodontal disease. Results were in the direction of overweight/obese women being more likely to have periodontal disease, although results were not precise enough to draw firm conclusions. This might be due to the high homogeneity of the study population - most of participants were not overweight, had a college or higher education, were employed, and were above the low income threshold – or it might indicate that the disparities seen in above studies that were mainly conducted in the United States and Brazil [9, 30, 31] are not apparent in our study population.

The only factor strongly associated with periodontal disease was perceived frequent bleeding during tooth brushing, even demonstrating a dose–response with the increased severity of the periodontal disease. Self-reported bleeding during tooth brushing was consistently associated with an increased risk of periodontal disease when applying the different periodontal disease definition of Arbes et al. and the US NHANES 2009 and 2010 in this study population (results not shown) [5, 23] This finding suggests in low-resource settings where there is shortage of dental human resources and the standard full-mouth dental examination is not routinely performed, self-reported frequent bleeding during tooth brushing could serve as a screening criterion for further periodontal disease examination by professionals.

Strengths of this study include reporting the prevalence of periodontal disease among a large sample of pre-conception women who were seeking care for reasons unrelated to oral health. The dental examination method used in this study was the standard full mouth examination. Limitations of this study include, first, given that our study was hospital-based, a convenient sampling process, the prevalence found in this population might not represent the all pre-conception women. Secondly, the participants had a high proportion of women with well-education and better social-economic status, which could limit the generalizability of findings, but might indicate that the general population at an even higher risk of periodontal disease.

## Conclusion

A high prevalence for periodontal disease of 73.9% was found among Chinese pre-conception women. Self-reported frequent bleeding during tooth brushing was significantly associated with the increased rate of periodontal disease, which could be regarded as a signal of requiring further oral health care. The results highlight the oral health care needs among Chinese pre-conception women. The screening and prevention of periodontal disease among pre-conception women should be included in maternal and child health care programs, in order to improve both maternal oral health and pregnancy outcomes.

## Abbreviations

BMI: Body mass index
CAL: Clinical attachment loss
PD: Probing depth
